# Porous Structure Properties of *Andropogon gerardi* Derived Carbon Materials

**DOI:** 10.3390/ma11060876

**Published:** 2018-05-24

**Authors:** Natalia Howaniec, Adam Smoliński

**Affiliations:** 1 Department of Energy Saving and Air Protection, Central Mining Institute, Pl. Gwarków 1, 40-166 Katowice, Poland; 2 Central Mining Institute, Pl. Gwarków 1, 40-166 Katowice, Poland; smolin@gig.katowice.pl

**Keywords:** biomass, char, porous structure, carbon material

## Abstract

Various carbonaceous materials are valuable resources for thermochemical conversion processes and for production of materials of proven sorption properties, useful in environmental applications for gaseous and liquid media treatment. In both cases, the parameters of the porous structure of carbon materials are decisive in terms of their physical and mechanical properties, having direct effects on heat and mass transport as well as on sorption capacity and selectivity. The physical activation of carbon materials produced from various precursors is widely discussed in literature. In this respect, the effects of temperature and partial oxidation of carbonaceous materials with steam or carbon dioxide are mostly considered. The reports on the effects of pressure on the development of porous structures of carbon materials are, however, extremely limited, especially when biomass as a precursor is concerned. In this paper, the results of an experimental study on the effects of pressure in the range of 1–4 MPa on the specific surface area, the total pore volume, average pore diameter, and microporosity of carbon materials prepared with the use of *Andropogon gerardi* biomass as a precursor are presented. The tested samples were prepared at the temperature of 1000 °C under an inert gas atmosphere in the high-pressure thermogravimetric analyzer. The most developed porous structure was reported for carbon materials produced under 3 MPa. The highest volume of narrow micropores was characteristic for materials carbonized under 2 MPa.

## 1. Introduction

Depletion of natural resources and increasing energy demand imply the need for implementation of more sustainable technologies in all aspects of economic development. In particular, this concerns recycling and utilization of waste materials but also the wider use of biomass as a raw material of low carbon footprint. Biomass valorization has been widely discussed recently in terms of its utilization in thermochemical processing technologies, including pyrolysis and gasification [1]. Gasification of biomass chars alone or in blends with fossil fuels or waste materials is also a subject of research, in particular when production of hydrogen as a clean energy carrier is considered [2,3,4,5]. Various biomass-derived materials and gasification residues are also valuable resources for the production of activated carbons of proven sorption properties, useful in environmental applications for gaseous and liquid media treatment [6,7,8,9]. In both cases, the parameters of porous structure of carbon materials determine their physical and mechanical properties, heat and mass transport processes, as well as sorption capacity and selectivity. The physical activation of carbon materials produced from various precursors is widely discussed in literature, where the effects of temperature, heating rate, activation time, and partial oxidation of carbonaceous materials with steam or carbon dioxide are mostly discussed [6,8,10,11]. Physical activation methods are often combined with chemical activation with various agents, e.g., KOH, NaOH, ZnCl_2_, H_3_PO_4_, (NH_4_)_2_S_2_O_8_ [7,9,12,13,14]. However, reports on the effects of pressure on the development of the porous structure of carbon materials are limited and concern mostly the characterization of coal chars’ behavior, including their reactivity, in gasification and combustion processes [15,16,17,18]. The studies presenting the effect of pressure on the development of biomass-derived carbon material porosity are even more scarce [19,20]. The pressures range applied in previous studies is also quite narrow and relatively low (0.5–2.5 MPa) [15,19], while the heating rates are high (of a few hundred °C per minute), representing the conditions of gasification reactors. Such conditions may not reflect the optimal terms for production of carbon materials of most developed or tailored porous structures. Therefore, in this paper, the results of the experimental study on the effects of physical activation with pressure in the range of 1–4 MPa, and at high temperature of 1000 °C on the development of porous structure of carbon materials prepared with the use of *Andropogon gerardi* biomass as a precursor are presented. The effect of pressure on the specific surface area, the total pore volume, average pore diameter, and microporosity of biomass-derived carbon materials is discussed. The results prove pressure to be a parameter of considerable potential in shaping the porous structure of carbon materials.

## 2. Materials and Methods

The biomass sample was acquired from an experimental stand of M&D Farms Sp. z o. o. in Swierczow, Poland. *Andropogon gerardi* is a grass of 1–2.5 m long blades cultivated mainly as an energy crop and animal feed crop, but is also applicable in anti-erosion and reclamation activities in post-industrial areas [21,22]. Its cultivation potential in climate conditions of central Europe is considerable, producing up to 24 tons of dry mass per hectare on moderately moist soils of pH 5–8. This makes it also a potential precursor for carbon materials of enhanced sorption properties. The ultimate and proximate analyses of the biomass and its ash composition analysis were performed in the accredited laboratories of the Central Mining Institute with the application of relevant standards. The results of these analyses are given in Table 1.

**Figure 1:**
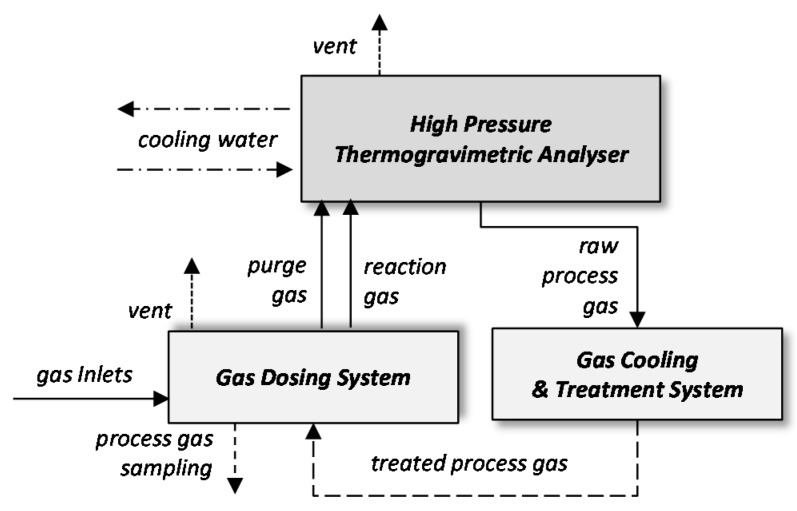
Schematic diagram of the installation for biomass carbonization at high pressure.

The biomass was carbonized with the use of a high-pressure thermogravimetric analyzer with the magnetic suspension balance mechanism (Rubotherm GmbH, Bochum, Germany), at the temperature of 1000 °C in an inert gas (Ar) atmosphere and under pressure of 1, 2, 3, or 4 MPa for 5 h (see Figure 1). A heating rate of 20 °C/min was applied. In each experiment, 1 g of biomass sample was used. The resultant chars were crushed and sieved below 0.2 mm, outgassed overnight at 120 °C and analyzed with the use of gas sorption analyzer Autosorb iQ (Quantachrome Instruments, Boynton Beach, FL, USA) [23]. The specific surface area was quantified on the basis of the nitrogen isotherm data at −196 °C and with the use of the multi-point BET method [24]. Pore size distribution of the micro-mesoporous material was determined with the use of the Density Functional Theory (DFT) method [25]. The total pore volume was determined as the volume adsorbed at the relative pressure of 0.99. The volume and area of micropores were computed with the use of V-t-deBoer method based on the nitrogen sorption isotherm at −196 °C data [26] and with the application of the Monte Carlo (MC) method and carbon dioxide isotherm at 0 °C [27]. The images of the carbon materials were taken by scanning electron microscopy (SEM) using a SU-3500N microscope (Hitachi High-Technologies Corporation, Tokyo, Japan).

## 3. Results and Discussion

**Figure 2:**
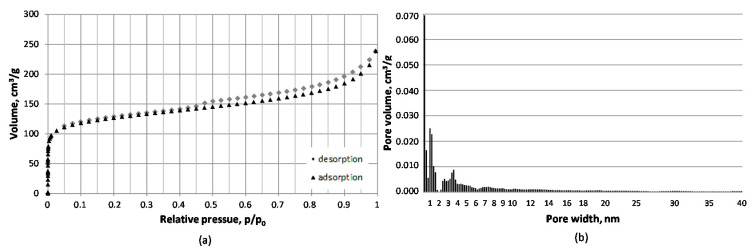
Nitrogen isotherm at −196 °C (**a**) and DFT volume histogram (**b**) for carbon material produced under 3 MPa and 1000 °C.

The results of the porous structure characterization are given in Table 2. The nitrogen sorption isotherms for all chars show the complex structure of the micro- and mesopores network reflected in the high uptake at low relative pressures, with microporous structures and hysteresis loops distinctive for mesoporous materials [23]. The shape of the hysteresis loop is typical for irregular structures and slit-like pores [23]. The exemplary isotherm for sample no 3 carbonized under 3 MPa is presented in Figure 2a. This char was proved to have the most developed porous structure in terms of the specific surface area, total pore volume, and micropore volume and area (see Table 2). The increase in carbonization pressure in the range of 1–3 MPa resulted in the significant increase in the specific surface area and the total pore volume. With the increase in pressure from 1 to 2 MPa, the specific surface area and the total pore volume doubled. The application of 3 MPa pressure resulted in the specific surface area and the total pore volume almost three times higher than for chars produced at 1 MPa (Table 2). Further increase in pressure to 4 MPa resulted in a decrease in the specific surface area and the total pore volume of about 19 and 23%, respectively, when compared to the maximum values reported for 3 MPa chars. This effect is in line with previous observations made for other precursors, including coal [15,16,17,18] and biomass [20], though the opposite tendency of the surface area of biomass-derived carbon materials to decrease with pressure has also been reported [19].

Micropore volume and area determined with the application of the V-t-deBoer method (pores width range 0.6–2.0) almost tripled with the increase in carbonization pressure from 1 to 3 MPa and slightly decreased from the maximum values (of about 14%) with further rise in pressure to 4 MPa. The data for micropore volume and area determined on the basis of the carbon dioxide isotherm (pore width range 0.4–1.5 nm) showed a 25% increase in micropore volume and 23% increase in micropore area with the pressure increase from 1 MPa to 2 MPa and a slight decrease of about 6% and 7%, respectively, with increase of pressure to 3 MPa, and of 6% with further rise in pressure to 4 MPa (Table 2). The variations in the average pore diameter of carbon materials produced under various pressures were not significant and within the experimental error (Table 2). The pores of the diameter of 0.57 nm contributed the largest share of the total pore volume according to the DFT model.

The DFT pore size distribution results showed the dominant role of microporosity (pores of a diameter below 2 nm), especially in the range of up to 1 nm, in shaping the pore volume of the carbon materials tested (see Figure 2b). Micropores contributed to approximately 49–56% of the total pore volume of chars. The next distinct pore diameter range visible in the volume histogram was 2–5 nm with the share of about 20% in the total pore volume. The remaining part of pores, approximately 27–34% of the total pore volume, comprised macropores of diameter over 5 nm (Figure 2b).

**Figure 3:**
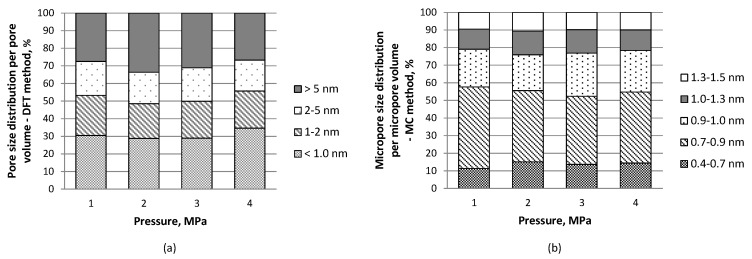
Pore size distribution per pore volume (**a**) and narrow micropore size distribution per narrow micropore volume (**b**) for carbon materials tested.

Pore size distribution within the entire pore width range measured based on the nitrogen sorption isotherm as well as narrow micropore distribution measured on the basis of the carbon dioxide isotherm data showed comparable shares of pores of particular diameter ranges in carbon materials volume, irrespective of pressure applied (Figure 3). However, the values of pore volumes and areas differentiated the carbon materials produced under various pressure conditions (Table 2).

**Figure 4:**
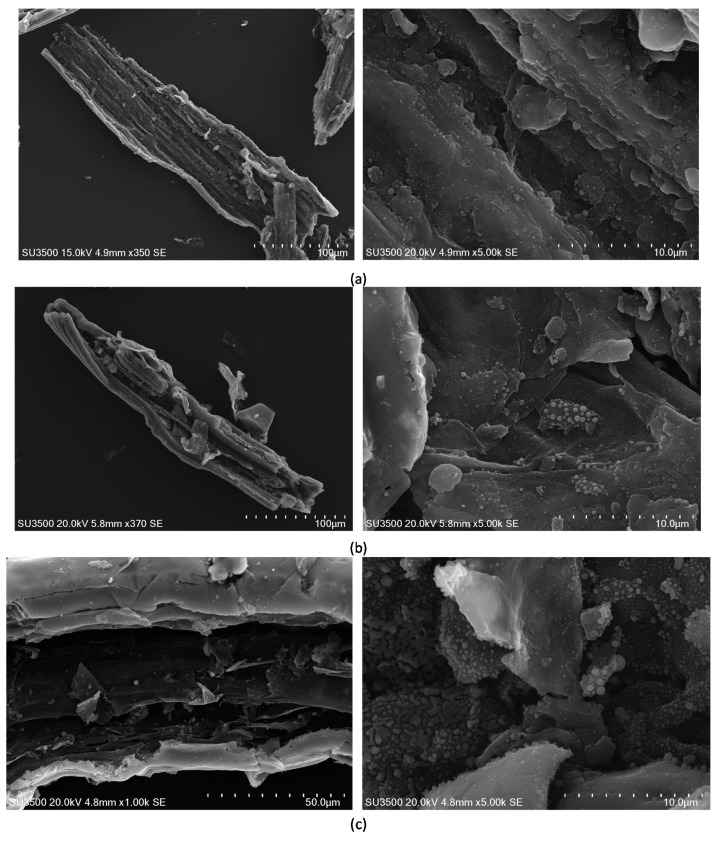

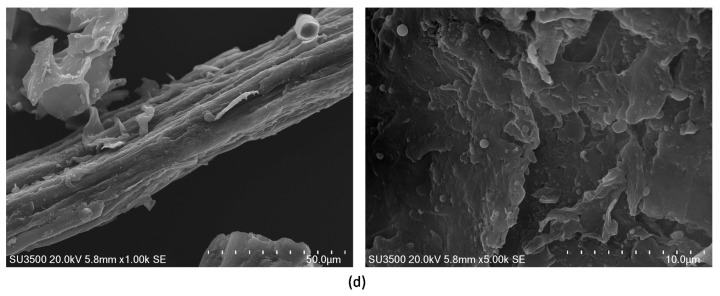
SEM images of carbon materials produced at 1000 °C and under the pressure of (**a**) 1 MPa, (**b**) 2 MPa, (**c**) 3 MP, and (**d**) 4 MPa.

The disintegration of carbon materials produced under pressure and at high temperature resulting mainly from moisture and volatiles release under low heating rates can be seen in Figure 4. The differences in particle shape between carbon materials produced under various pressures are slight. All the samples maintained the parent material cell shape, while demonstrating slits and openings, which seem to become more complex with the pressures applied from 1 to 3 MPa (Figure 4a–c). It can be also noticed that increasing the pressure in the range of 1–3 MPa resulted in raising the number of smaller particles visible in cavities created by the devolatilization. The carbon materials produced under 4 MPa showed a smoother surface than the particles created under lower pressures (Figure 4d). This may be the effect of fusion of the previously melted particles under the final external stress of the high pressure applied. The surface of the cavities is also smoother and poor in smaller particles.

The dominant role of micropores in the porous structure of chars generated under high pressure might imply that relatively low heating rates applied in this study enabled free swelling behavior of chars, which enhanced the development of microporosity. Intensive melting behavior, previously observed to be more pronounced with high heating rates applied in carbonization step, would lead to the development of macroporous materials [18]. The slight decrease in the development of porous structure under the pressure of 4 MPa reflected in the decreased specific surface area and the total pore volume, as well as micropore area and volume, may also be the effect of merging of previously melted particles under the increased external stress. The pressure of 3 MPa may, therefore, be considered as the threshold value under the experimental conditions applied, exceeding which would result in the counteracting effects of the pressure on the porous structure development with the release of moisture and volatiles (Table 1) as well as swelling behavior of the carbonized material [18,19,20]. If the target is to receive a carbon material with the largest volume of narrow micropores, then the value of 2 MPa can be considered optimal on the basis of the carbon dioxide isotherm data (Table 2).

The results observed proved that the pressure may be considered as an effective parameter shaping the porous structure of carbon materials, especially with lower heating rates. Similar values of the specific surface area as well as micropore area and volume were reported for the more complex and costly process of physical and chemical activation of peat with carbon dioxide at 750 °C and acid washing [8]. Comparable values of the specific surface area, micropore volume, and total pore volume to presented here were also obtained with chemical activation by impregnation with H_3_PO_4_ of cotton stalks (ratio of 0.3:1) followed by thermal activation at 500 °C for 2 h [14]. Carbon materials produced from hazelnut shells by mixing with citric acid, autoclaved at 250 °C for 7.5 h, and then thermally treated at 600 °C for 2 h also showed similar values of the specific surface area and the total pore volume to the ones reported here [7].

This proves that pressure may be considered as a practical tool for production of porous materials from various precursors, including non-lignocellulosic biomass, known to be less applicable in activated carbon production. It may replace, at least to some extent, the time-consuming and labor-intensive processes of chemical and physical activation. Clearly, the application of pressure itself is not sufficient to provide materials of the specific surface area and total pore volume comparable with commercial activated carbons or the materials after a complex cycle of physical and chemical activation of most suitable precursors [6,8,12,14]. Nevertheless, its application was proved to produce wood- and grass-derived materials with porous structures that are more developed than those obtained by the chemical activation of waste biomass, e.g., rice husk [9,20].The relatively low heating rates applied in this study also resulted in higher values of the specific surface area (of 40%) and micropore area (of 60%) than previously observed for pine-derived carbon materials produced under 2 MPa with high heating rates of 500 °C/min [19]. This implies that low heating rates are more applicable in tailoring the porous structure of carbon materials for high microporosity.

## 4. Conclusions

In this paper, the results of an experimental investigation of the effects of pressure on high temperature carbonization of *Andropogon geraradi* biomass were presented, enabling the following conclusions to be drawn:

1. Carbonization of the selected biomass under 3 MPa gives carbon materials with the most developed porous structure in terms of the specific surface area, the total pore volume, and microporosity, as determined by nitrogen sorption isotherm;
2. The pressure of 2 MPa was proved to enhance the development of narrow microporosity, as quantified by carbon dioxide isotherm;
3. Application of pressure during carbonization enables tailoring of the porous structure of carbon materials.

## Figures and Tables

**Table 1:** Physical and chemical properties of *Andropogon gerardi* biomass.

Parameter, Unit	Value
**Proximate Analysis**	
Total moisture, % *w/w*	9.72
Ash, % *w/w*	3.87
Volatiles, % *w/w*	70.26
Fixed carbon, % *w/w*	16.15
**Ultimate Analysis**	
Sulfur, % *w/w*	0.06
Carbon, % *w/w*	53.3
Hydrogen, % *w/w*	7.57
Nitrogen, % *w/w*	bdl
Oxygen, % *w/w*	25.54
**Heating Value**	
Lower heating value, kJ/kg	14,242
**Ash Composition**	
SiO_2_, % *w/w*	14.67
Al_2_O_3_, % *w/w*	3.18
Fe_2_O_3_, % *w/w*	0.93
CaO, % *w/w*	37.10
MgO, % *w/w*	3.46
Na_2_O, % *w/w*	0.53
K_2_O, % *w/w*	22.13
SO_3_, % *w/w*	4.12
TiO_2_, % *w/w*	0.15
P_2_O_5_, % *w/w*	13.17
ZnO, % *w/w*	bdl

**Table 2:** Parameters of the porous structure of carbon materials determined on the basis of nitrogen sorption isotherm at −196 °C and carbon dioxide sorption isotherm at 0 °C.

Sample No.	Pressure, MPa	Multi-Point BET, m^2^/g	Average Pore Diameter, nm	Total Pore Volume, cm^3^/g	V-t-deBoer Volume, cm^3^/g	V-t-deBoer Area, m^2^/g	MC Volume, cm^3^/g	MC Area, m^2^/g
1	1	162.16	3.29	0.133	0.046	113.37	0.146	456.68
2	2	333.23	3.25	0.271	0.102	248.29	0.182	561.85
3	3	468.70	3.15	0.369	0.116	285.70	0.171	523.13
4	4	380.46	2.98	0.283	0.100	249.78	0.160	492.76
